# Visual Detection and Image Processing of Parking Space Based on Deep Learning

**DOI:** 10.3390/s22176672

**Published:** 2022-09-03

**Authors:** Chen Huang, Shiyue Yang, Yugong Luo, Yongsheng Wang, Ze Liu

**Affiliations:** 1 Automotive Engineering Research Institute, Jiangsu University, Zhenjiang 212013, China; 2 State Key Laboratory of Automotive Safety and Energy, Tsinghua University, Beijing 100084, China

**Keywords:** automatic parking, uneven lighting, visual detection, deep learning, image processing

## Abstract

The automatic parking system based on vision is greatly affected by uneven lighting, which is difficult to make an accurate judgment on parking spaces in the case of complex image information. To solve this problem, this paper proposes a parking space visual detection and image processing method based on deep learning. Firstly, a 360-degree panoramic system was designed to photograph the vehicle environment. The image has been processed to obtain a panoramic aerial view, which was input as the original image of the parking space detection system. Secondly, the Faster R-CNN (Region-Convolutional Neural Network) parking detection model was established based on deep learning. It was aimed to detect and extract the parking space from the input image. Thirdly, the problems of uneven illumination and complex background were solved effectively by removing the background light from the image. Finally, a parking space extraction method based on the connected region has been designed, which further simplified the parking space extraction and image processing. The experiment results show that the mAP (mean Average Precision) value of the Faster R-CNN model using 101-Floor ResNet as the feature extraction network is 89.30%, which is 2.28% higher than that of the Faster R-CNN model using 50-Floor ResNet as the feature extraction network. The model built in this paper can detect most parking spaces well. The position of the output target box is accurate. In some test scenarios, the confidence of parking space recognition can even reach 100%. In summary, the proposed method can realize the effective identification and accurate positioning of parking spaces.

## 1. Introduction

In recent years, visual detection has been the research trend of artificial intelligence, especially in the direction of autonomous driving. When detecting distance with radar in traditional parking [1,2,3], there must be a reference vehicle on both sides of the parking space. Furthermore, the position and angle of parking are strictly required. To solve the above problems, visual parking systems [4,5] have been proposed. During these years, many scholars have completed extensive research in the field of vision detection parking systems.

Target detection has been widely studied. It is mainly designed for specific targets, such as face recognition and pedestrian detection. However, it is not easy to detect other targets [6,7,8]. To address this issue, Suhr et al. [9] proposed an end-to-end single-stage parking space detection method, which used Convolutional Neural Network (CNN) to simultaneously obtain global information and local information, and combined them with the attributes of the parking space to detect the parking space. Dusan et al. [10] utilized active visual ID tags to simultaneously locate and identify vehicles. The tags with 2-D color can be captured by cameras above the parking lot, thus effectively protecting against changes in ambient lighting and helping to reduce costs. To solve the problems of low recognition rate and weak generalization ability brought by vision-based parking space detection methods, Xu et al. [11] proposed a deep convolutional neural network-based parking space detection method. This method used the improved fish-eye image to directly detect the parking spaces in the fish-eye image through the YoloV3 network structure. Yu et al. [12] proposed a detector using a Convolutional Neural Network (CNN) to obtain faster speed and smaller model size while maintaining the detection accuracy of parking space. Bandi et al. [13] used multiple surveillance cameras to obtain real-time information. The parking spaces were detected and classified by the Mask R-CNN model. However, the method used here was just a simulation. The hardware setup has not been performed.

In terms of visual algorithm, Zhang et al. [14] modeled multiple optimal pedestrian detectors based on existing features, such as HOG and LUV. They used real-boost and other learning methods to generate new features. The result showed that it was accurate but costly to identify. Similarly, Zhou [15] utilized a probabilistic target recognition algorithm to detect road markings and used a particle filter to track lane markings, which was aimed to realize the day and night detection and tracking of road markings. However, it relied on the inertial sensor to send tracking lane marking information, which was not suitable for any situation. On this basis, Raghuraman [16] proposed a new method to detect lane markers by replacing the probabilistic target recognition algorithm with a robust enhancement algorithm. The results of his experiment showed that the algorithm was effective in both white and night scenes.

At the same time, image processing technology has been an urgent problem to be solved for automatic parking. Wang et al. [17] combined the images acquired by the camera into an aerial view by using the Levenberg–Marquardt image Mosaic algorithm. The feature of the parking space was extracted by Radon transform. The automatic parking was realized through double arc trajectory planning and preview control strategy. Shen et al. [18] utilized the MRF model to generate a new visual-spatiotemporal path based on a pair of vehicle images. Similarly, Li et al. [19] proposed a Deep Joint Discriminant Learning (DJDL) model to solve problems related to vehicle image features.

However, the above-research fails to consider that visual automatic parking is susceptible to uneven lighting, especially in the case of complex image environment information, which makes parking space recognition very difficult. To solve the problems mentioned above, this paper designs a parking space detection and image processing method based on deep learning. 

The research contents of this paper mainly include the following points. Firstly, four fisheye cameras were designed as a 360-degree panoramic system. The function of this system is to provide the original input images for training and testing for building a parking space detection system based on deep learning. Secondly, two parking space detection models based on deep learning were built and trained. In total, 3644 training and testing samples containing valid annotated parking spaces have been produced. They were used for model training and testing. Additionally, typical scenes such as complex lighting conditions and night mode have been tested. Finally, an image processing method has been designed. The image was preprocessed. The RGB image was converted into a grayscale image. The grayscale image was denoised to reduce the interference of noise on parking space recognition. Operations, such as binarization, extraction of connected regions, and morphological processing, were performed. A Hough transform was used to detect the straight line of the image obtained after morphological processing. The processing of the parking space line in the image and the vertex location of the parking space have been completed. The coordinates of the vertices of the parking spaces obtained by the positioning were converted into the panoramic image to complete the final positioning work.

**Figure 1:**
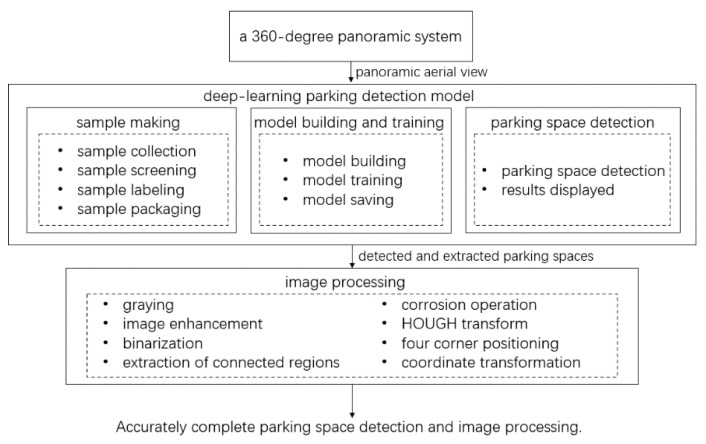
Overall flowchart of the proposed technique.

The technical route of this paper is shown in Figure 1.

## 2. Models and Methods

### 2.1. Fish-Eye Camera Model

**Figure 2:**
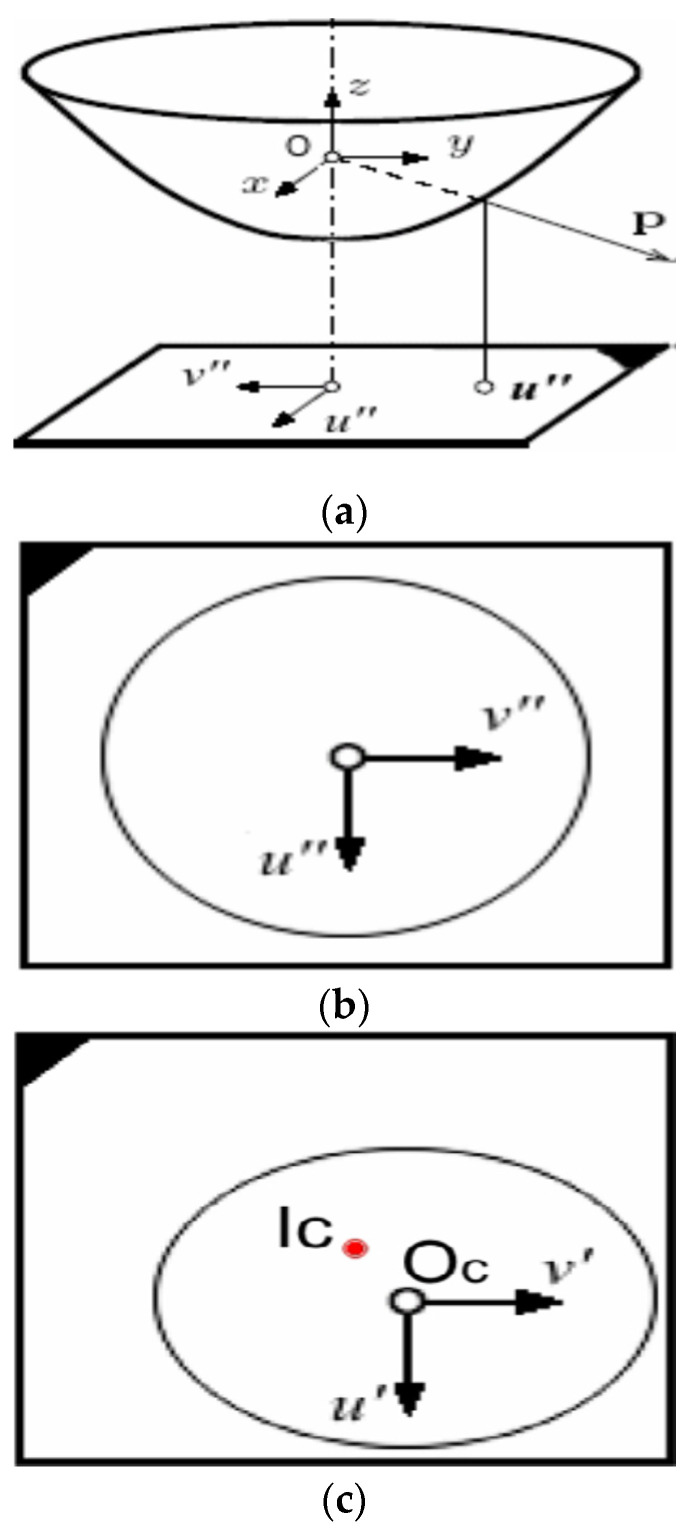
Fish-eye camera model and coordinate system. (**a**) Camera model. (**b**) Imaging plane coordinate system. (**c**) Image coordinate system.

In this paper, four fisheye cameras [20] are designed as a 360-degree panoramic view system, which can collect image information around vehicles. The model description of fisheye cameras is shown in Figure 2.

In this model, the projection system from world coordinates to image plane coordinates is a non-linear projection function containing camera distortion parameters. Taylor series is utilized for polynomial expansion, as shown in Formula (1).

(1) $$f\left(\rho \right)=a_{0}+a_{1}\rho +a_{2}\rho^{2}+\ldots +a_{n}$$

In the formula, $\;\rho =\sqrt{u^{\prime \prime \;2}+v^{\prime \prime \;2}}$ is the distance between the projection point and the center of the imaging surface; $a_{0},a_{1}\ldots \ldots a_{n}$ is the coefficient of the Taylor series expansion polynomial and the camera distortion parameter.

$Rt=\left[r_{1},r_{2},r_{3},t\right]$ is utilized to represent the rotation translation matrix from world coordinates to camera coordinates. The projection relation between point P(x,y,z)  in world coordinates and its imaging point is as follows:

(2) $$\lambda P=\lambda \left[\begin{array}{c} u^{\prime \prime }\\ v^{\prime \prime }\\ f\left(\rho \right) \end{array}\right]=Rt\cdot P=\left[r_{1}\;r_{2}\;r_{3}\;t\right]\cdot \left[\begin{array}{c} x\\ y\\ z\\ 1 \end{array}\right]\;\;$$

There is an affine transformation relationship between the image point coordinates and the pixel coordinates on the image, as shown in Formula (3):

(3) $$\left[\begin{array}{c} u^{\prime \prime }\\ v^{\prime \prime } \end{array}\right]=\left[\begin{array}{cc} c & d\\ e & 1 \end{array}\right]\cdot \left[\begin{array}{c} u^{\prime }\\ v^{\prime } \end{array}\right]+\left[\begin{array}{c} uc\\ vc \end{array}\right]$$

### 2.2. Deep-Learning Parking Detection Model

**Figure 3:**
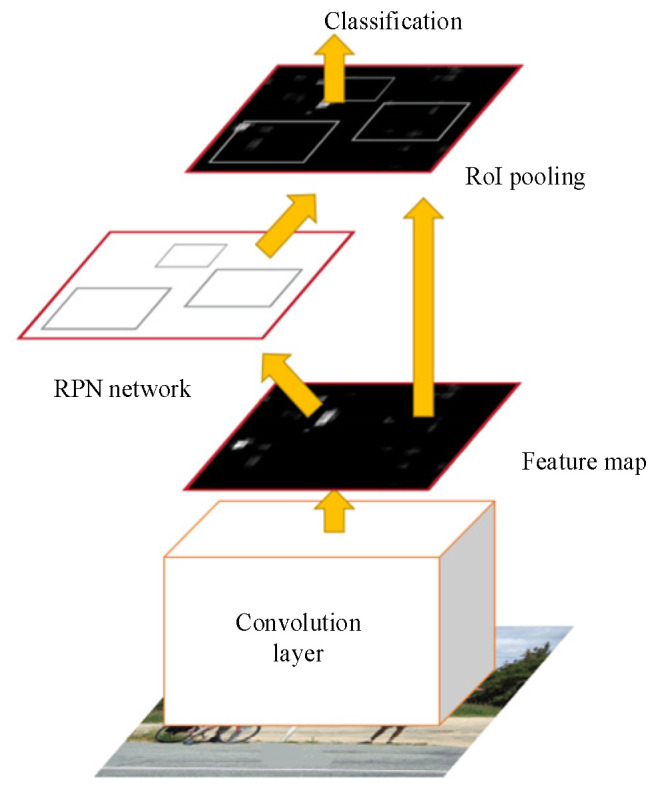
Faster R-CNN model structure.

Compared with traditional models, the Faster R-CNN model not only improves the training and testing speed greatly but also takes a short time to detect images in real time. Therefore, the Faster R-CNN [21,22] is selected as a parking detection model in this paper, as shown in Figure 3. The running processes of the model are as follows:

(1) Firstly, a feature layer can be obtained by using the convolutional neural network to extract the features of the input original image;
(2) Secondly, the RPN network replaces the traditional selective search algorithm to nominate candidate regions;
(3) Thirdly, the nominated area is judged to contain the target. The location and size of the target box are adjusted as well;
(4) Finally, further regression adjustment is carried out for the candidate area classification and target box position and size.

**Figure 4:**
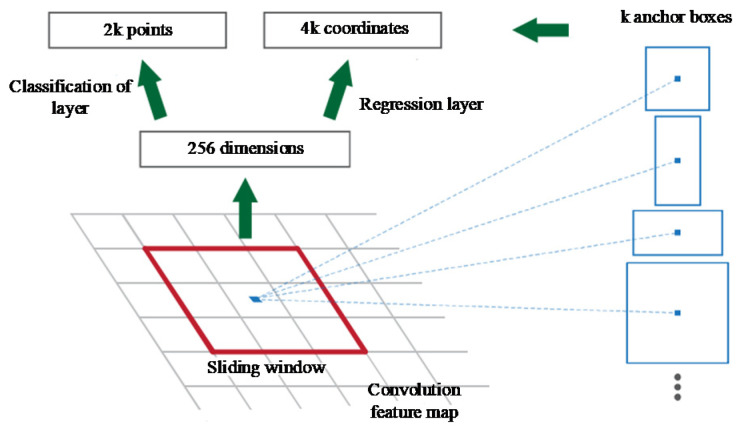
RPN model.

Specifically, the faster R-CNN model designs an RPN (Region Proposal Network) model [23]. This can replace the traditional selective search algorithm for the nomination operation of the candidate region, as shown in Figure 4. The front end of the model uses a convolutional neural network for feature extraction and takes the obtained feature layer as the input of the RPN network. RPN network proposes the concept of an anchor. It takes each pixel of the input feature map as a base point to generate K candidate boxes. The usual value of K is 9. The candidate window is shown on the right side in Figure 4. A square rectangle with two directions and the candidate-zoom boxes of these three basic graphs are generally set to 0.5 and 2 for scale. Finally, 9 candidate boxes are obtained. Classification and regression operations are carried out based on the candidate boxes. The classification operation is to judge the generated candidate box. It can determine whether there is a target in the candidate box and output 2k points. The regression operation refers to the translation and scaling of the position of the candidate frame. It can also mean the regression of the position and width of the border. Four times k coordinate values can be output through the operation. Then, the appropriate candidate area can be extracted through judgment. The candidate boxes are mapped to the convolution layer by using the RoI operation, which is similar to the Fast R-CNN model. Finally, classified and boundary regression operations are performed.

### 2.3. Image Processing 

#### 2.3.1. Graying 

An image is a matrix composed of a pixel. Image processing is to manipulate the pixel matrix. For color images, the color of a pixel can be represented by Formula (4), where (*x*, *y*) represents the coordinates of the pixel, and any color can be represented as the linear sum of three basis vectors. Therefore, the corresponding colors of the specific pixels can be changed by determining the specific coordinates of the pixels and changing the values of the three-color channel corresponding to the pixels.

(4) $$C\left(x,y\right)={\left[R\left(x,y\right),G\left(x,y\right),B\left(x,y\right)\right]}^{T}={\left(R,G,B\right)}^{T}$$

In Equation (4), C(x,y) represents the color of the pixel point at (x,y) coordinates. R(x,y) represents the red channel value at the pixel with coordinates (x,y). G(x,y) represents the green channel value at the pixel with coordinates (x,y). B(x,y) represents the value of the blue channel at the pixel with coordinates (x,y). It can be seen from the formula that after the coordinates of the pixel point are determined, the color of the pixel point can be changed by changing the RGB channel value corresponding to the coordinates of the pixel point.

With the model trained by deep learning, some small areas can be detected and extracted from the panoramic aerial view. These areas contain images of only one parking space, which are raw RGB color images. To process color images, we need to adjust the RGB values separately, which greatly increases the computation. At the same time, RGB images cannot represent morphological features, but image colors. Therefore, RGB color images should be converted into grayscale images to reduce the amount of calculation in image processing. Graying [24] refers to changing the values of the three basis vectors *R* *G* *B* of the pixel points color matrix to make them equal, which is *R* = *G* = *B*.

(5) $$R_{1}=G_{1}=B_{1}=0.3R+0.59G+0.11B$$

There are three commonly used grayscale methods: the maximum value grayscale method, the average value grayscale method, and the weighted average grayscale method. Although the maximum grayscale method retains the best details, it is easy to cause the image to be too bright locally and interfere with the recognition. The image details obtained by the average method are well preserved, but there is still a lot of noise in the image. In contrast, the weighted average method retains the details better. This method can best remove noise, and can better control the local overbrightness. In this paper, the weighted average method was selected for grayscale processing. The optimal parameters were obtained after experiments. Its expression is shown in Equation (5).

#### 2.3.2. Image Enhancement 

**Figure 5:**
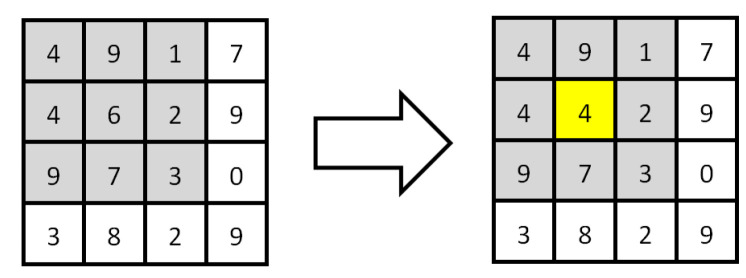
Median filtering.

There will be a lot of noise in the image because the camera will be disturbed by many factors when taking photos. It will interfere with the identification of parking spaces. The region of interest in the image is enhanced to reduce noise interference and improve the accuracy of recognition. That is to preserve the details of the image and suppress the noise as much as possible, which is necessary to filter. The filtering effect will directly affect the subsequent processing and recognition. In this paper, spatial domain enhancement is selected because it is more suitable for parking detection. Median filtering [25] is better than Gaussian filtering in detail retention and image noise suppression. After a comprehensive comparison, median filtering is finally selected. The principle of median filtering is to use a filter template of a certain size to average the region of a certain size in the image, as shown in Figure 5. A 3 × 3 filter template is utilized as an example. The filter template is composed of the pixels to be processed and the 8 pixels around them. The concept of the fuzzy radius is utilized in median filtering. The fuzzy radius of the 3 × 3 template is 1. The gray value of the pixel to be processed is calculated from the gray value of each pixel in the template. For example, the 9 pixels on the left are calculated to be worth 5. It is taken as the new coordinate of the central pixel point of the filter. The median filtering operation can be realized after the whole picture is traversed.

#### 2.3.3. Binarization

Threshold segmentation should be carried out on the image before parking space recognition. The foreground and background should be separated, which is called binarization [26]. The most basic threshold segmentation method is global binarization. It obtains the global threshold through the gray level calculation of the whole image. The pixels higher than this threshold are set to 255. The pixels lower than thresh are set to 0. The formula is shown in (6), where *x* represents the gray value of the input pixel, *f*(*x*) represents the gray value of the calculated output pixel, and *T* is the threshold value of binarization. The maximum inter-class variance method OSTU [27] is used to calculate the optimal threshold *T*.

(6) $$f(x)=\left\{ \begin{array}{l} 0\;\;\;\;\;\;\;\;\;\;\;\;\;\;\;\;\;x<T\\ 255\;\;\;\;\;\;\;\;\;\;\;\;\;x>T \end{array}\right.$$

#### 2.3.4. Extraction of Connected Regions

**Figure 6:**
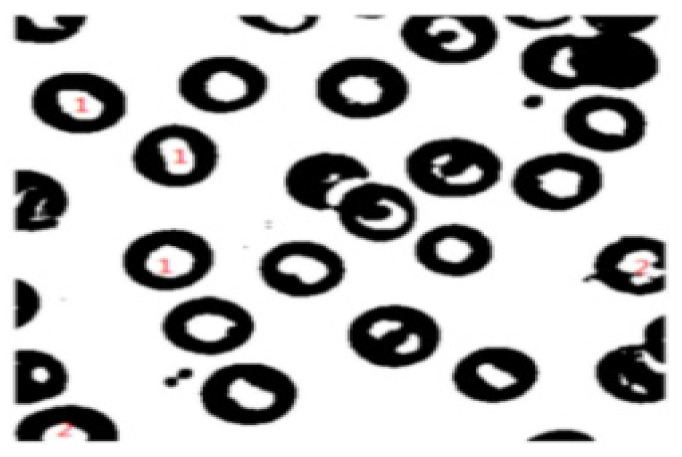
Holes in a binary image.

**Figure 7:**
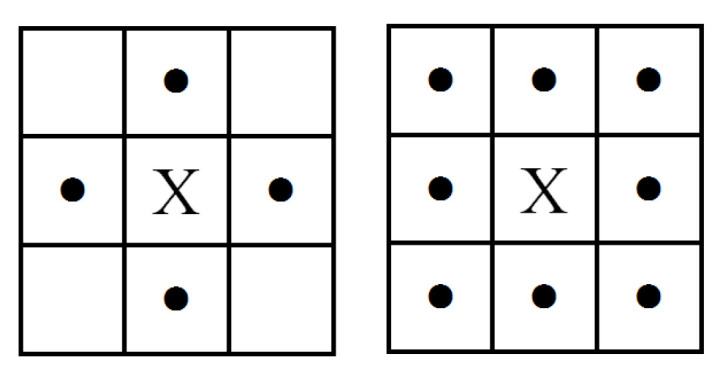
Four-neighborhoods and eight-neighborhoods.

To eliminate the interference of jumble straight lines and facilitate the positioning of the four vertices of the parking space, this paper designs a method based on the connected area to extract the parking space. The hole filling of the binary image is introduced [28] before extracting the connected region, as shown in Figure 6. The white feature is surrounded by the black feature (black is the closed feature) in the area marked 1 in the figure, which is called a closed hole. The white part is surrounded by the area marked 2, which is called an open hole. The extraction method is to fill the closed-hole area in the image first, and then realize the extraction of the connected region. The four pixels, (*x*, *y* − 1), (*x*, *y* + 1), (*x* − 1, *y*), and (*x* + 1, *y*), adjacent to the vertical and horizontal of a pixel X are assumed to be M4(X). The four pixels, (*x* + 1, *y* + 1), (*x* + 1, *y* − 1), (*x* − 1, *y* + 1), and (*x* − 1, *y* − 1), adjacent to the diagonal of pixel X are assumed to be MD(X). Four-neighborhoods are represented by M4(X). The union of M4(X) and MD(X) is called 8-neighborhoods. It is called 4 adjacencies if the gray value of 4 pixels in the 4 neighborhoods is equal to the gray value of center point X. Similarly, it is called 8 adjacencies if the gray value of 8 pixels in the 8 neighborhoods is equal to the gray value of the center point X pixel point. This definition can be represented in Figure 7. Pixel A and pixel B are connected if pixel A and pixel B are 4-adjacency relations. Furthermore, pixel A and pixel C are also connected if we assume that pixel B and pixel C are 4-adjacency relations. Thus, the 4-connected pixels can form one block together, which is called the 4-connected region. The 8-connected region can be defined in the same way.

#### 2.3.5. Corrosion Operation

The obtained connected region usually contains small edge burrs and other irrelevant regions. These can be solved by the open operation in morphological processing [29]. Open operation is a combination of corrosion and expansion operations. In other words, the image is corroded first, and then the same size structural elements are utilized to expand the corroded image [30].

Certain structural elements are used by the corrosion operation to erode the edges of objects. The result of the corrosion is to reduce the area by a circle if the image contains an area larger than the structural element. The area will break or disappear after the corrosion operation if the image contains an area smaller than the structural element. The corrosion operation is expressed by Formula (7), where (*x*, *y*) is the coordinates of the pixel; *Θ* is a symbol for corrosion operations; *A* is the area to be processed; and *B* is the structural element for corrosion operation.

(7) $$A\Theta B=\{x,y|{\left(B\right)}_{xy}\subseteq A\}$$

After the corrosion operation, the small burrs in the connecting area will be eroded. However, the contour of the whole area also shrinks one circle at the same time. To solve this problem, the main area needs to be restored to its original size through expansion operation. The formula of expansion operation can be expressed as:

(8) $$A⊕B=\{x,y|{\left(B\right)}_{xy}\cap A\neq \emptyset \}$$

In this formula, (*x*, *y*) is the coordinates of the pixel; ⊕ is the expansion operation symbol; *A* is the area to be processed; *B* is the structural element of the expansion operation.

**Figure 8:**
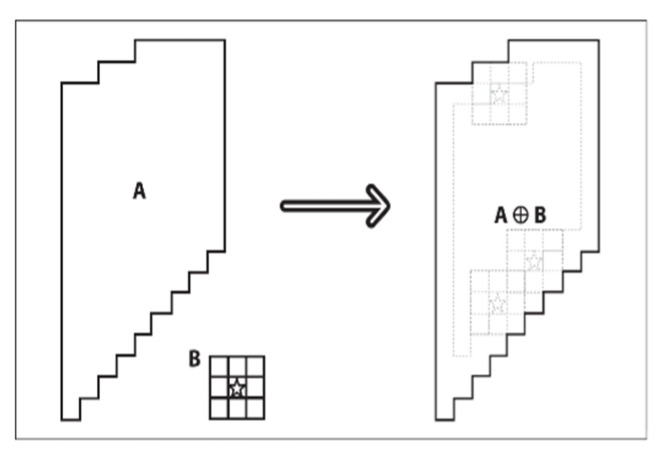
Expansion operation diagram.

The structural element *B* is utilized to traverse and move in image *A*. The pixel point at (*x*, *y*) is assigned a value of 1 if the intersection between structural element *B* and the coincidence area is not empty when the origin of *B* moves to (*x*, *y*). Otherwise, it is assigned a value of 0. After all the traversal, the expansion operation is completed. The diagram is shown in Figure 8.

#### 2.3.6. Hough Transform

**Figure 9:**
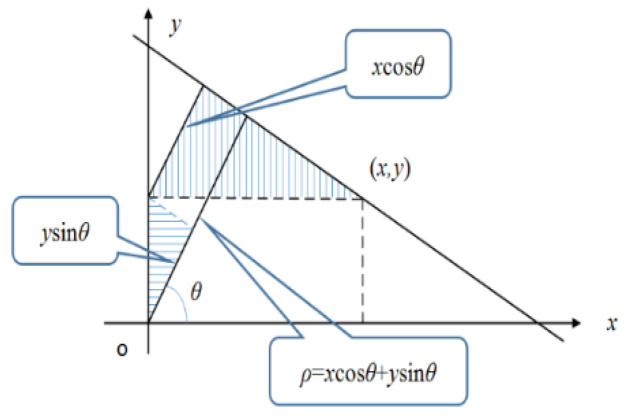
Cartesian and polar coordinates.

Four vertices need to be positioned after the pre-selected parking space is obtained. In this paper, an improved Hough transform method is adopted to make the decision. Hough transform [31] is a method to transform the straight-line detection problem into the point detection problem in the polar coordinate system. Cartesian and polar coordinates can be converted. Under the rectangular coordinate system of a straight line corresponds to the polar coordinates of ρ  and θ. Conversion methods are shown in Figure 9. In the figure, θ  is the angle between the line perpendicular to the line and the *X*-axis. ρ is the distance between the origin and the given line.

**Figure 10:**
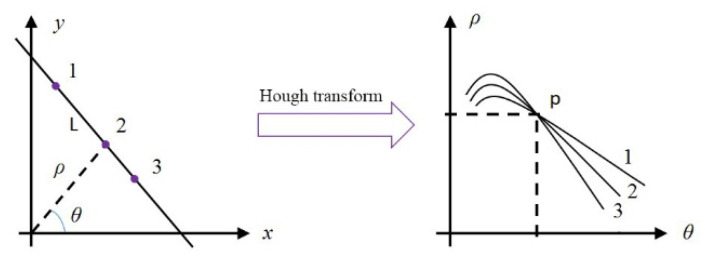
Hough transform schematic.

As shown in Figure 10, there are an infinite number of straight lines passing through a point $\;\left(x_{1},y_{1}\right)$ in the rectangular coordinate system. Each of these lines corresponds to a point (ρ,θ) in polar coordinates. That corresponds to a sine curve $\;\rho_{1}=x_{1}cos\theta +y_{1}sin\theta$ in polar coordinates if we convert all lines to polar coordinates. Similarly, all lines that go through point $\left(x_{2},y_{2}\right)$ in cartesian coordinates correspond to another sine curve $\;\rho_{2}=x_{2}cos\theta +y_{2}sin\theta$ in polar coordinates. These two sinusoids have an intersection. The curve of the polar coordinate system will produce many coincidence points after traversing each pixel of the whole image. We accumulated the number of overlapping points and counted the number of pixels that each line passes through. Finally, the detection of the line has been completed.

**Figure 11:**
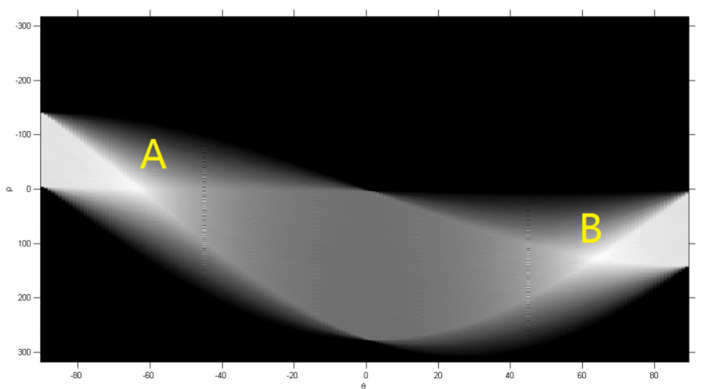
Hough line detection.

**Figure 12:**
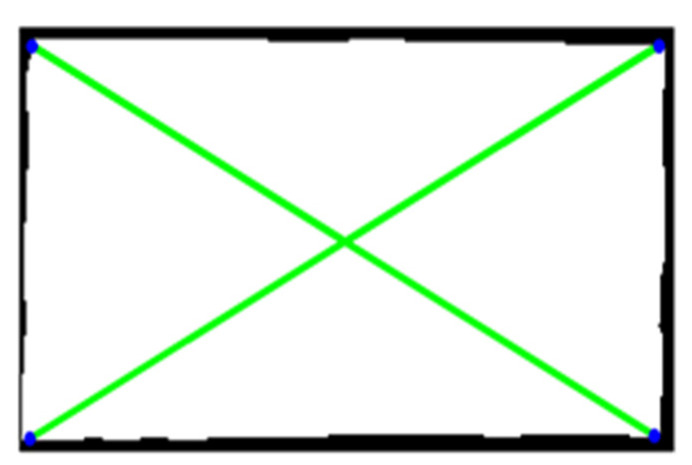
Location diagonal detection.

We obtained the line detection schematic diagram after the Hough transformation. As shown in Figure 11, the abscissa and ordinate of the image are, respectively, A and B. The brighter the point in the image, the more effective pixel the line passes through. The two brightest points in the figure are point A and point B. They, respectively, correspond to the two longest line segments in Figure 12. That is two diagonal lines. The four-corner positioning of the pre-selected parking spaces has been finally realized. These four vertex coordinates can form a parallelogram through calculation. At the same time, the area meets the requirements of the parking area. This area can be determined as a parking space. The coordinate output of four vertices has been completed as well. The positioning is accurate. It can be used for automatic parking.

#### 2.3.7. Location Coordinate Transformation 

The coordinates of four vertices of the parking space can be obtained after using the Hough transform for line detection. Moreover, these coordinates need to be converted to coordinate values corresponding to the 360-degree panoramic image. It is assumed that the coordinates corresponding to the detected region are (*X*_0_, *Y*_0_, *W*_0_, *H*_0_), where *X*_0_ is the horizontal coordinate corresponding to the upper left corner of the detected region in the 360-view image, and *Y*_0_ is its corresponding vertical coordinate, *W*_0_ is the width corresponding to the extracted region, and *H*_0_ is the height corresponding to the extracted region. In addition, it is assumed that the coordinates of a parking vertex q point located by the designed algorithm in the extracted image are (*x*, *y*). Then, the coordinates converted to a 360-degree panoramic view system are assumed to be (*X*, *Y*). The following coordinate transformation relationship is as follows:

(9) $$\{\begin{array}{c} X=x+X_{0}\\ Y=y+Y_{0} \end{array}$$

## 3. Experiment and Results

At present, there are many deep learning development platforms. This paper chose TensorFlow as the experimental platform for deep learning. The construction, training, and identification of the network were all implemented using this platform. TensorFlow is a deep learning framework developed by Google. The framework has the characteristics of high flexibility and good portability. It is one of the most popular [32] development platforms in the field of deep learning.

Due to the large amount of data generated during the training process of the deep learning model for parking space detection. It is easy to overflow the memory and cause the model to fail to train. So the memory capacity was upgraded to 12 GB size. A large number of convolution operations are used in the model, and the CPU is slow to run deep learning. Model training often takes months. This experiment used the GeForce GTX 1060 graphics card to accelerate the training of the built deep learning model. Training time was reduced to less than 20 days. The specific hardware device configuration information is shown in Table 1. 

**Figure 13:**
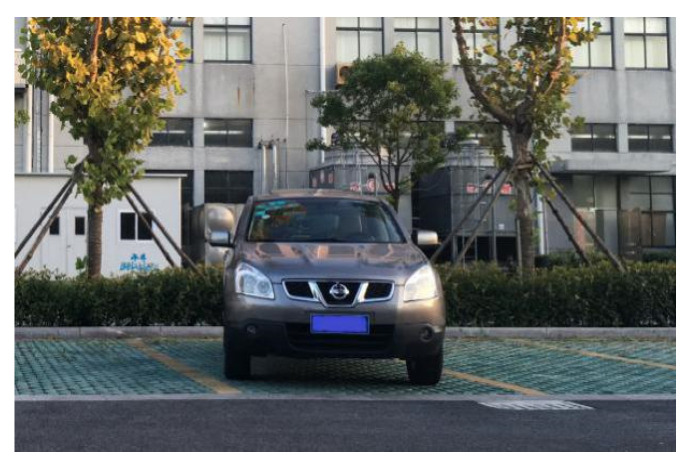
Test vehicle.

This test vehicle with a 360-degree surround view system is equipped with four 180-degree wide-angle fisheye cameras. These cameras are fixed to take pictures around the vehicle. The test vehicle is shown in Figure 13. 

To begin with, this system was used for shooting parking spaces in different scenes and under different lighting conditions. In total, 1300 frames of effective images have been collected. 

**Figure 14:**
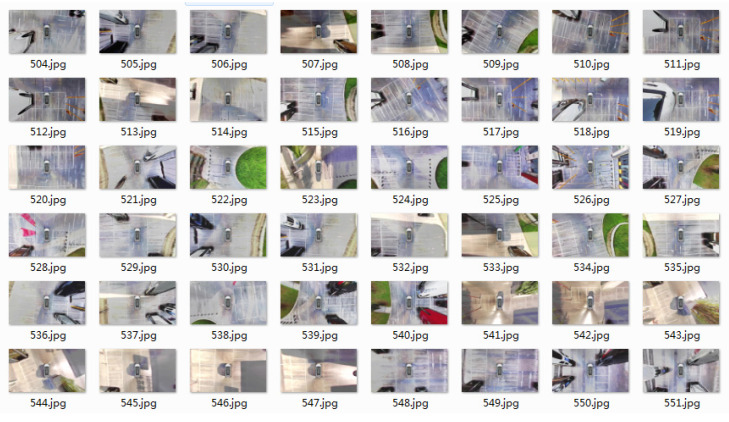
Valid images.

Then, the established deep learning parking space detection model was used to detect and extract parking spaces from valid images. The samples were trained and tested as well. As shown in Figure 14, these images contain a total of about 10,000 parking spaces. 

**Figure 15:**
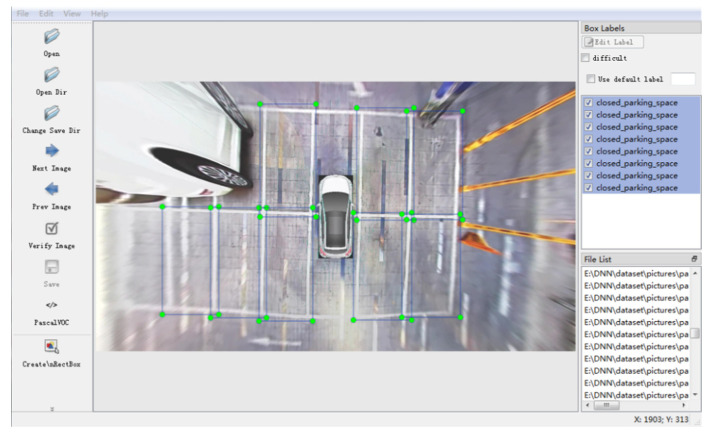
Parking space identification.

Furthermore, the collected images were annotated to determine the target of model training. It was aimed to specify the specific location of the parking space in the image, as shown in Figure 15. In this experiment, 1300 pictures were marked and 3644 parking spaces with effective marks were obtained. 

Finally, the samples were divided according to the ratio (7:3). In total, 2550 were randomly selected as the training samples of the test model. The rest have become test samples of the model.

Model training was conducted in 200,000 steps for the Faster R-CNN model. We used 50-layer RESNET and 101-layer RESNET, respectively, as the feature extraction network. To intuitively display the effect of the model after training, the mean average precision (mAP) was used as the evaluation index of the model effect [33]. 

AP is the average precision rate of a single tag (the average of the maximum precision rate in each recall rate). The calculation method of the average precision rate of a single tag AP is as follows: assuming that there are M positive samples in N samples. M recall values can be obtained (1/M, 2/M, …, M/M). For each recall value r, the corresponding maximum precision rate (r’> r) can be calculated. Then, the average of these M precision values can be obtained to obtain the final AP value. The mAP is the average accuracy rate of all class tags. In other words, it is the average of the mean accuracy AP values for all categories. 

Among them, the precision rate refers to how many of the predicted positive samples are positive samples. The formula of the precision rate is as follows:

(10) $$Precision=\frac{TP}{TP+FP}$$

*TP* represents the correct prediction of the number of positive samples. *FP* represents the number of positive samples that were incorrectly predicted. 

Recall indicates how many positive samples were correctly predicted out of all samples. The formula is as follows:

(11) $$Recall=\frac{TP}{TP+FN}$$

*TP* is the correct prediction of the number of positive samples. *FN* is the number of negative samples that were incorrectly predicted.

**Figure 16:**
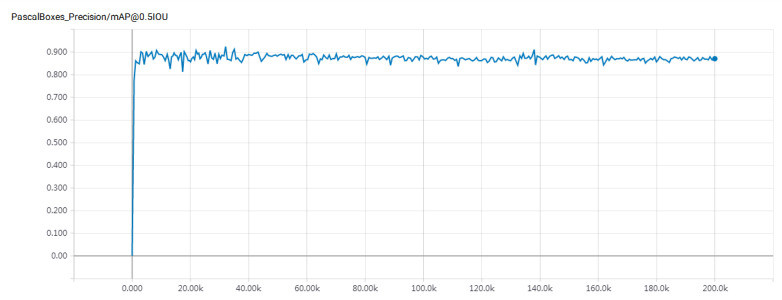
MAP value of faster R-CNN (50-layer ResNet) model.

**Figure 17:**
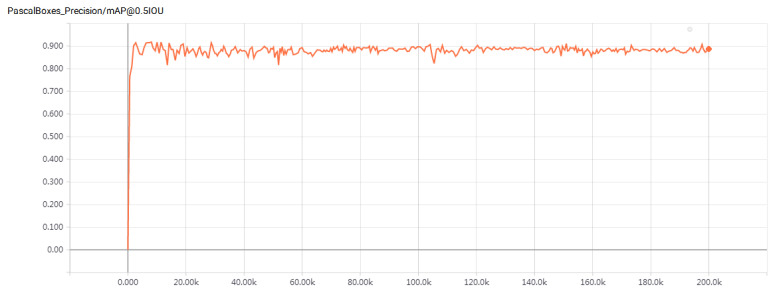
MAP value of faster R-CNN (101-layer ResNet) model.

**Figure 18:**
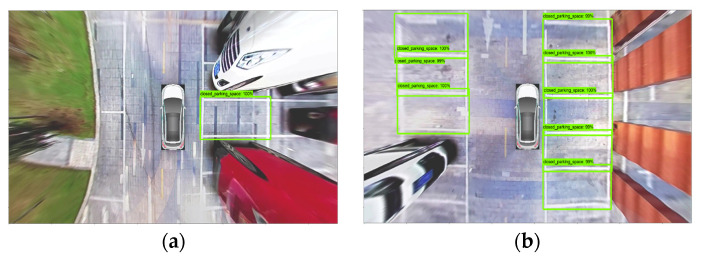

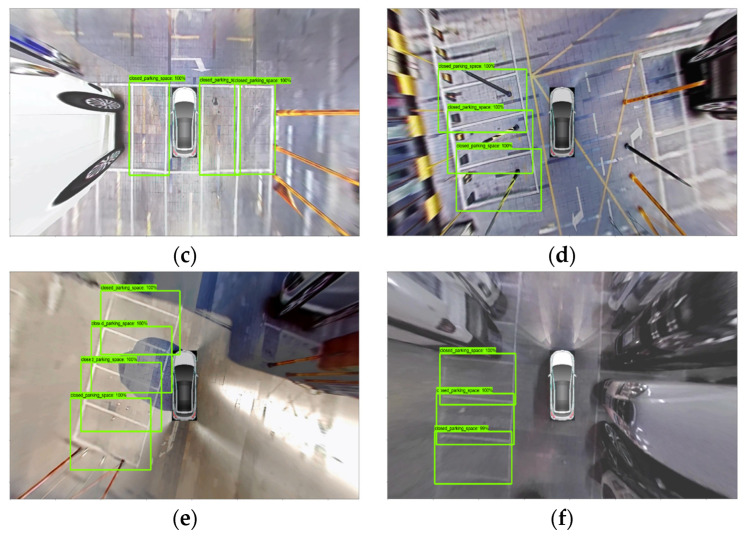
Test results. (**a**) Scene with fewer parking spaces. (**b**) Scene with lots of parking spaces. (**c**) Side parking scene. (**d**) Complex scene with ground marking lines. (**e**) Scene with complex lighting environment and lots of shadows. (**f**) Night scene.

The experimental results are shown in Figure 16 and Figure 17. In comparison, the Faster R-CNN parking detection model using 101-Floor ResNet as the feature extraction network has a better detection effect. Its mAP value has reached 89.30%, which is 7.30% higher than the mAP value of the model of Bazzaza et al. Bazzaza et al. used the architecture of the YOLOv4 Deep Learning network to detect available parking spaces [34]. Therefore, the faster R-CNN parking detection model using 101-Floor ResNet as the feature extraction network has been used to detect different parking spaces. The results of the test are shown in Figure 18.

Figure 18a is a scene with one complete parking space. There are two incomplete special parking spaces on the left. Since the training sample has only a few parking spaces of this type, its characteristics have not been learned and thus were not detected. This type of parking space training sample can be added in subsequent studies. Thus, the recognition rate of this type of incomplete parking space can be improved.

Figure 18b shows that the scene contains eight complete parking spaces. The experiment detected parking spaces with clear parking lines. Additionally, it has detected the parking spaces with fuzzy parking lines, such as the three parking spaces on the left. It is difficult for traditional image detection. 

Figure 18c simulates the side parking scene, in which there is a large white area interference on the left side of the figure. For traditional images, such a situation will cause a great interference to binarization in the image. It is easy to cause the failure of the algorithm. Luckily, the model established in this paper is not affected by the problem. As can be seen from the figure, three complete parking spaces have been detected. The confidence level has reached 100%.

Figure 18d is a scene in which the ground sign line is complex and the angle between vehicles and parking spaces is relatively large. For this scene, the parking space detection method based on traditional images is difficult to complete the processing. The parking spaces with less occlusion have been detected. However, there are three parking spaces on the right, which have not been detected due to the influence of relatively fuzzy and nearby sign poles. In this case, the model can be improved by adding more pictures of this scene to the training sample of the model;

Figure 18e shows a scene with a complex lighting environment and a lot of shadows. It is very difficult to solve for traditional images. However, the model built in this paper can detect the parking spaces well. All of them are included in the marquess, with confidence levels up to 100%.

Figure 18f shows the night scene with very low light, which can also obtain good detection results.

**Figure 19:**
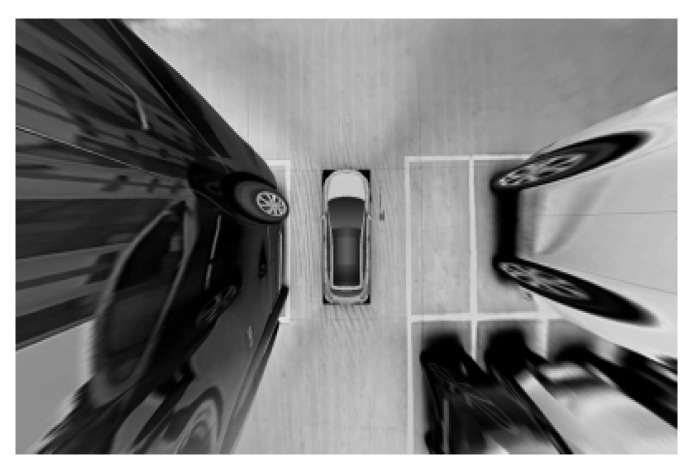
Grayscale and filtering.

**Figure 20:**
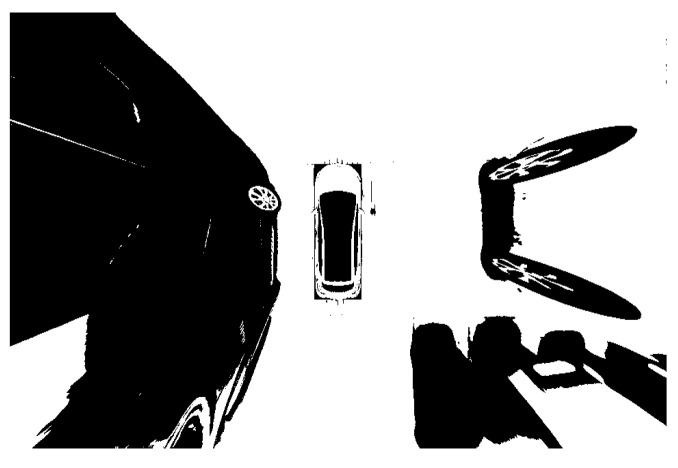
Binarization.

**Figure 21:**
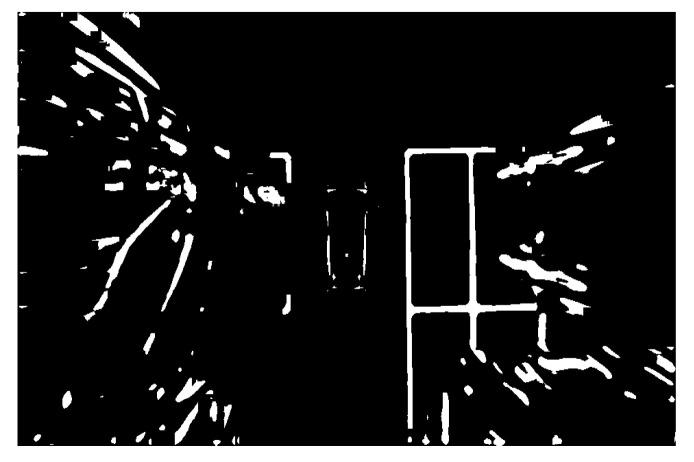
Background light removal.

The image processing has been carried out on the detected parking space to further identify and locate the parking space. The results are shown in the following figures. Figure 19 is the image after grayscale and filtering. It indicates that the over-bright state has been well controlled. Additionally, the image noise has been significantly reduced. The parking space and other details have been well preserved. Since the processed image is still illuminated unevenly and the image contains a lot of interference information, such as many unrelated vehicles. The image should be binarized and the background light should be removed. As shown in Figure 20 and Figure 21, the stop line disappears after binarization. After removing the background light, the parking space has been more complete.

**Figure 22:**
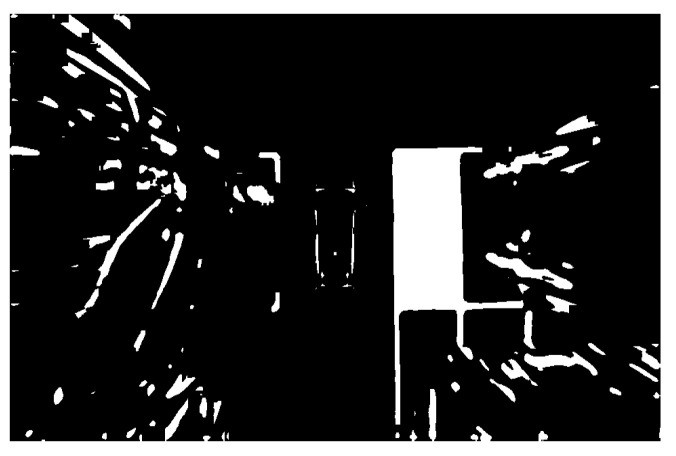
Connected region extraction.

**Figure 23:**
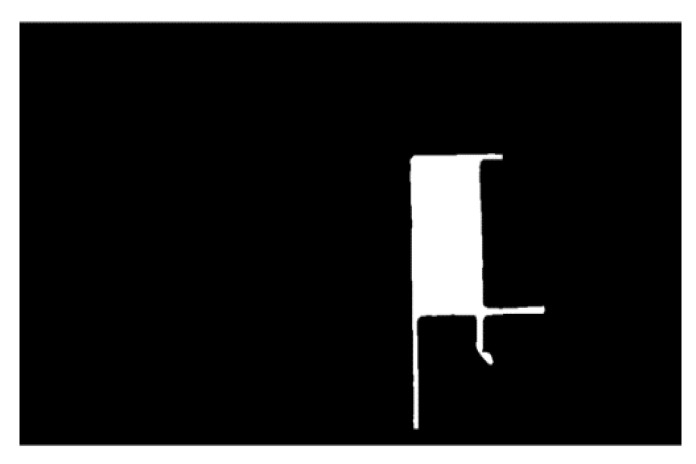
Corrosion operation.

**Figure 24:**
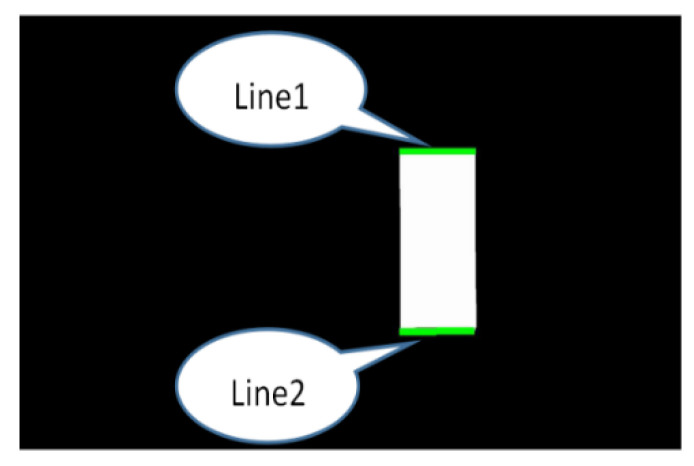
Upper and lower sides of the parking spaces.

**Figure 25:**
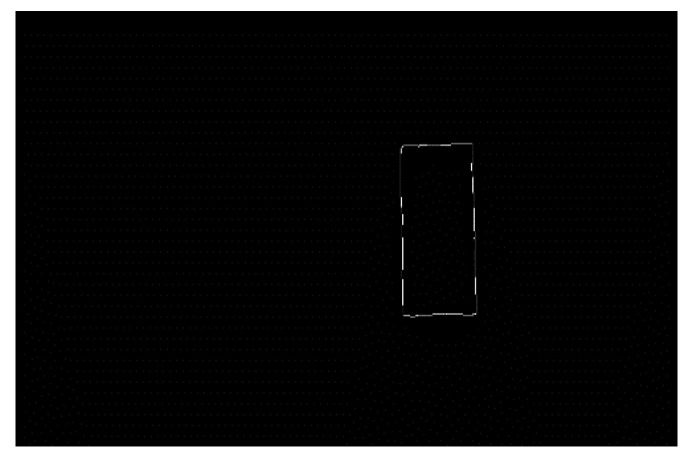
Hough transform.

**Figure 26:**
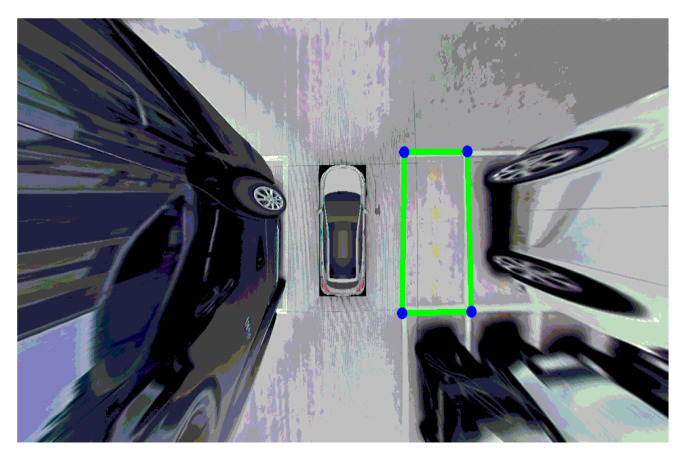
Coordinate transformation.

Moreover, the closed-hole area in the image has been filled. The connected area in the image has been extracted as well. In addition, the areas unrelated to the parking space have been corroded through the calculation of corrosion and expansion, as shown in Figure 22 and Figure 23. To simplify the calculation, the range of the Hough line detection angle has been limited to −23°~23°. The upper and lower sides of the parking spaces have been detected by the Hough transform, as shown in Figure 24. In addition, the four vertex coordinates of the parking space were obtained by the Hough transform. These coordinates were transformed into coordinates corresponding to the 360-degree panoramic image, as shown in Figure 25 and Figure 26. Finally, the connected regions extracted were determined as parking spaces by the geometric relationship of the four vertices. Experimental results show that the proposed method can accurately detect and recognize parking spaces in the case of uneven illumination or complex background, which complemented the work after parking space detection in the paper by Bandi et al. [13].

In this paper, the sensitivity analysis of the built model has been carried out on the parameters of the clarity of the parking space line, the proportion of the white area in the image, and the angle between the vehicle and the parking space. The experimental results are shown in Table 2.

The model can still maintain a high recognition rate for the parking space when the definition of the parking space line is low. However, when the clarity of the parking space line is lower than 32%, the recognition rate of the model for the parking space will be significantly reduced. For traditional models, when there is a large white area in the image, it will cause a great interference to the binarization, which will cause the algorithm to fail. The model built in this paper can still maintain a high recognition rate in the face of large white area interference. Only when the proportion of white areas reaches 61% will it have a relatively large impact on the recognition rate of the model. The angle between the vehicle and the parking space has little effect on the recognition rate. Regardless of the angle between the vehicle and the parking space, the model can maintain a high level of recognition rate for the parking space.

## 4. Conclusions

In this paper, a fisheye camera was used to take real-time pictures of the surrounding environment of the vehicle. At the same time, a faster R-CNN parking detection model was established, which could detect and extract parking spaces from images as the image input of the parking positioning system. Additionally, we addressed the inability of global binarization to handle images with uneven lighting or complex backgrounds by removing background light from the original image. A parking space extraction method based on connected regions was proposed. This method simplified the extraction of parking spaces. It removed interference from irrelevant areas. Finally, the identification and positioning of parking spaces have been realized. The experimental results show that the proposed method can accurately complete the tasks of parking space detection and image processing in the case of uneven illumination or complex background.

However, there are still some deficiencies, which need further research and improvement. On one hand, the parking space detection and positioning system designed in this paper was based on the detection of parking space lines. Therefore, it does not affect the system output of the parking space when there are obstacles in the parking space. To meet the application-level automatic parking requirements, multi-sensor fusion can be considered in the follow-up research. On the other hand, due to hardware limitations, the only graphics card we have cannot load both the training program and the test program. The training program was placed on the graphics card for training. The test program was carried out on the CPU. Since the test program greatly occupied the memory and CPU computing resources, the training speed of the GPU has been slowed down. The training process lasted 190 h. In future deep learning research, it is possible to consider adding two graphics cards and running the test program on another graphics card to accelerate training. The model designed in this paper also has scenes that cannot be dealt with. For example, the parking space with serious damage to the parking space line and too vague parking space cannot be positioned. This is a difficult problem to solve and can be used as the direction of follow-up research.

## Figures and Tables

**Table 1:** Deep learning hardware platform information.

Hardware Equipment	Model Specifications	Amount
graphics card	NVIDIA GeForce GTX 1060 (6G VRAM)	1
CPU processor	Intel Core i5-3470	1
memory stick 1	Kingston DDR3 (8G memory)	1
memory stick 2	Kingston DDR3 (4G memory)	1

**Table 2:** Model sensitivity information.

Parameter	Sensitive Interval
parking line clarity	0–32%
the proportion of white area	61–100%
the angle between the vehicle and the parking space	/

## Data Availability

The data provided in this study are not public because they are provided by third-party car companies, which involve trade secrets and have not been publicly authorized by the car companies.
